# Extent of Reason in the Lower Animals

**Published:** 1891-03

**Authors:** A. McLaughlin


### EXTENT OF REASON IN THE LOWER ANIMALS.

BY A. MCLAUGHLIN, D. V. S.

The above title was the subject of a paper read before the "Association for the Study of Comparative Pschycology," by its President, Prof. McEachran, of McGill University, Montreal, in which he expressed the opinion that the lower animals must be possessed of a complicated mental state allied to our own in order that they may perform the many undoubted intellectual feats which they sometimes do ; in other words they must and do reason.

Other papers on the same subject, by the President, and different members, were very interesting. They all insist that the lower animals are not confined to "instinct" but have, "reason." Now I would like to ask, "who doubts it? what scientific man is there who claims that they do not reason?" The question to my mind should be, not as to whether they have reason, but to what extent they are capable of reasoning.

Instinct, according to Webster, "is a certain power or distinction of mind, by which, independent of all instruction, or experience, without deliberation, and without having any end in view, animals are unerringly directed to do spontaneously whatever is necessary for the preservation of the individual."

This definition confines instinct to a very small compass, and allows for reason a relatively large one, the definition for which might read, "a certain power or distinction of mind, by which, *dependent* on instruction or experience, *with* deliberation, and *with* an end in view, animals perform various acts.

I remember gazing, with a great deal of interest, when young, on a picture in my geography, of the monkeys in Brazil forming a natural bridge with their bodies, by which great numbers of them would cross a river.

Surely these monkeys had a very *plain end in view*, and they *very deliberately* acted to attain that end. It might be argued, that if the monkeys had not such long tails they could not possibly perform such a feat, and instinct, *unerring instinct*, taught them the use of those members as a means to escape their enemies. This argument would make instinct a very complex thing, indeed, so complex that the building of houses for protection from the inclemency of the weather might also be called a more highly developed instinct than that possessed by the monkey, but not greater in proportion as compared with other animals much lower in the grade of intelligence. The popular distinction between instinct and reason seems to be simply this, and it leaves no room for argument. The former is the intellectual faculty of the lower animals, the latter is the intellectual faculty of man.

Have you ever taught a puppy? Have you noticed the evident pleasure it gives him, when he realizes that he has *learned his lesson*, and when you advance him to another, and several lessons, how he watches you with each new trick you try to instil into his brain, his mental processes in a state of tension, anxious to comprehend what you are trying to teach him, but which his small mental calibre cannot as yet perceive; how in his efforts to do, what he does not yet understand, he does all the tricks he is already familiar with, and when he, at last, succeeds in mastering his last lesson, is he not pleased as well as yourself? Is this instinct? What has your lesson to do with the preservation of the individual or the species; of what use to him as a dog, is the ability at your bidding, to balance himself on a cup, jump through a hoop, etc? Is his evident pleasure, at his *knowledge* that he has pleased you due to instinct?

Here is a collie dog at my feet, as I write, who is thoroughly house-broke. If instinct is "unerring for the preservation of the individual" (which it is not) it is surely instinct to empty the bladder, the bowels, and to vomit, in the most convenient places; but this animal, like thousands of others, will not do either in the house, but instead will walk to the door, and stand there, knowing someone will open it. Does anyone pretend that *instinct* teaches that dog that it has a human being to wait on it? No, it may be instinct to imitate, but it is reason to say as plain as a boy in school, please let me go out, or I will be obliged to soil the floor. It might be argued that the dog is afraid of being punished but is not fear of punishment, a very human reason for doing many acts? But the dog does not always fear corporal punishment, you have taught him it was not right to do it, and for that reason he does not. The dog is a remarkably sensitive animal (at least some breeds); he is even capable of feeling ashamed, right and wrong—as it is taught him. Conscience is the ability to perceive that certain acts are wrong; it has nothing to do with what is right. What certain acts are depends entirely on what we have been taught, and on our surroundings; it is the intelligence of *fear*. It is the dog's training which forms his conscience; it is the same which forms our own. If my dog soils the floor it slinks away from me; *it knows it has done wrong*.

Does a dog understand spoken language? I have often heard this question asked. He knows a whistle, and a chirp; a terrier knows the word, rats! a setter, charge! others will answer to come here! drop it! beg! recognize their name, etc.

I tried the following experiment one day with a collie pup, not over two months old. I had taught him to "bring my gloves," and I wished to see if he would recognize the words. I spoke them in the middle of a conversation with my wife, as though the words were part of another sentence, and did not change my tone in the slightest, my back was toward the dog, but the *instant* I said the words, he left his play, ran into the next room where I had purposely hidden the gloves, and brought them back. I did this several times, and always with the same success. On one occasion as the little fellow was running out of the room on the same errand, he stopped at the door—stopped short—what is it I am going for, I have forgotten? was as plainly expressed in that moment's halt, as it is possible for an attitude to express a thought; so apparent was the act, that my wife and myself simultaneously expressed ourselves to that effect.

A cocker spaniel was brought to my office one day, with which my dog began playing, but which absolutely refused to join her, but as soon as her owner said, "go and play with the dog," she began romping with a right good will.

Almost any dog can be taught to beg, and how many will beg when they see you eating, plainly asking you to share your goodies.

I saw a little terrier a few days ago standing outside his own door, barking with a right good will. He wanted to get in, that was evident, and this was his method of attracting the attention of the inmates.

Just think what a complicated act that is. He knew his own house, he knew how to enter it, and the door being shut, he knew how to make his presence known. Under similar circumstances could man reason better, or act more to the purpose?

I have said a dog recognizes a chirp and a whistle; he does more; he knows when that chirp or whistle is *not* meant for him, for when I chirp to my horse my dog pays no attention, better than this, if I call another dog, she will help me catch him, as this incident, which happened several times, will prove: Some time ago I had a pup whom I was in the habit of giving an airing on a common, close by. The little fellow would invariably run on the grass, where I could not follow; I always tried to stop him by calling him back, but he never paid any attention; after a time, the larger dog who accompanied us would run after him, when I called, stand over him, and detain him until I caught him.

Dogs have more than instinct, they have, most decidedly, the powers of reason, and dogs are not classed as the highest order of intellect among the lower animals. To say they have not reason is an error, similar to another the laity indulge in, that Nature is a kind and good mother to them, and takes the best of care of them, that that unerring instinct, prevents them indulging in pernicious habits, and preserves them from being subject to those ills with which we poor humans suffer.

I look with interest to the proceedings of the Association; the members are discussing a study that is brimful of good to our profession.

